# Liquid nitrogen-treated autograft combined with megaprosthesis versus total femoral replacement for large-volume malignant femoral bone tumors: a comparative study

**DOI:** 10.1007/s00402-026-06432-y

**Published:** 2026-08-12

**Authors:** Nguyen Tran Quang Sang, Nguyen Duc Trung, Le Duc Huy, Luu Huu Phuc, Vu Duc Thang, Luong Nhat Anh, Le The Hung, Tran Thi Thu Hien, Tran Tuyet Thanh Hai, Duong Quang Vinh, Tran Van Cong, Nguyen Quoc Dat, Dang Minh Quang, Tran Duc Thanh, Tran Trung Dung

**Affiliations:** 1 Sarcoma Center, Vinmec Times City, Vinmec International Hospital, Hanoi, Vietnam; 2 Department of Orthopedic Surgery, College of Health Sciences, VinUniversity, Hanoi, Vietnam; 3 Department of Anesthesia and Pain Management, Vinmec Times City, Vinmec International Hospital, Hanoi, Vietnam; 4 Department of Surgery, Vinmec Phu Quoc, Vinmec International Hospital, Phu Quoc, An Giang Vietnam; 5 Institute of Biomedical Data and Science Research (BIODAS), Hanoi, Vietnam

**Keywords:** Osteosarcoma, Limb-salvage surgery, Liquid nitrogen-treated autograft, Megaprosthesis, Total femoral replacement

## Abstract

**Background:**

Limb-salvage surgery for extensive femoral osteosarcoma presents major reconstructive challenges. Conventional options such as total femoral replacement (TFR) and long-segment biological reconstructions are associated with significant complications. We evaluated the clinical outcomes of a novel hybrid approach combining a liquid nitrogen-treated autograft with a megaprosthesis (LN-MP) and compared them with those of TFR.

**Materials and methods:**

This retrospective study included 28 patients (range 8–24 years old) with malignant femoral bone tumors treated between 2020 and 2024. Patients underwent either LN-MP (n = 10) or TFR (n = 18). Perioperative outcomes, functional results (Musculoskeletal Tumor Society score—MSTS), bone union, oncological control, and survival were analyzed.

**Results:**

LN-MP demonstrated better functional outcomes (MSTS 27.0 ± 2.1 vs. 24.4 ± 2.2, p = 0.014), shorter operative time, and lower blood loss compared with TFR. Length of stay and follow-up duration were comparable. Continuous disease-free survival was higher in the LN-MP group (90% vs. 44%, p = 0.058), with lower recurrence (10% vs. 28%). Bone union was achieved in 70% of LN-MP cases at a mean of 9 months, with longer graft length associated with delayed union.

**Conclusions:**

LN-MP reconstruction may serve as a joint-preserving alternative to total femoral replacement for patients with extensive malignant femoral tumors. In this preliminary series, LN-MP was associated with favorable functional outcomes, satisfactory graft-host bone union, and acceptable oncological results. Given the retrospective design, limited sample size, and short follow-up duration, further studies with larger cohorts and longer follow-up are required to define the role of LN-MP among contemporary limb-salvage reconstructive strategies.

**Level of evidence:**

Therapeutic, Level III: retrospective comparative cohort study.

**Graphical abstract:**

**Figure:**
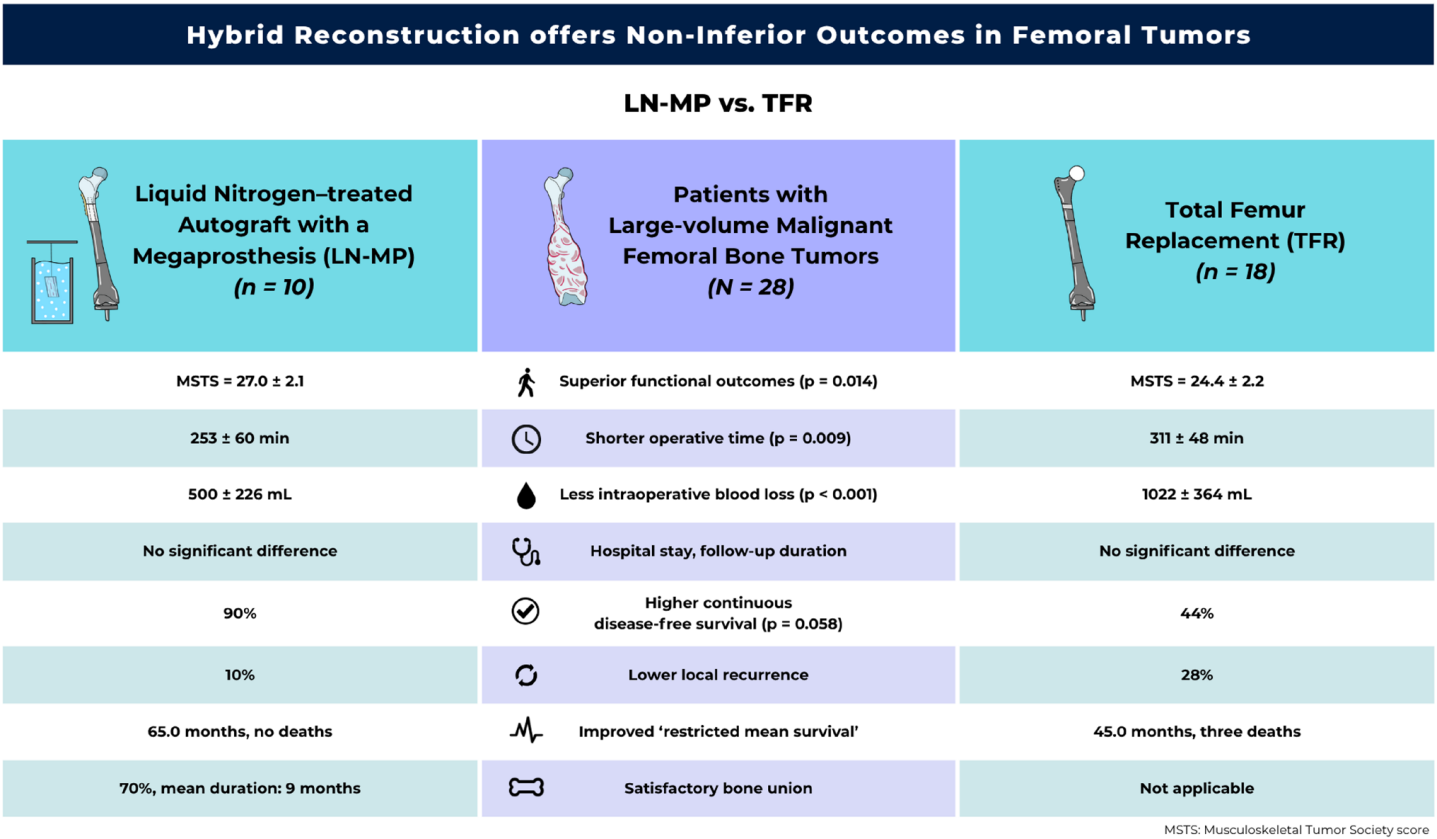

## Introduction

Limb-salvage surgery is now performed in the vast majority of extremity osteosarcoma cases [1]. The most frequent locations of bone sarcoma are the distal and proximal femur, the proximal tibia, and the proximal humerus. Reconstruction following tumor resection generally includes three approaches: endoprosthetic replacement, biological reconstruction (e.g., autograft or allograft), or composite techniques (allograft-prosthesis composite, autograft-prosthesis composite). Endoprostheses allow early mobilization but are associated with complications such as loosening, infection, and high cost [2]. In contrast, biological reconstruction offers long-term durability through bone regeneration, although techniques such as vascularized autografts are technically demanding and carry donor-site morbidity [3].

In cases of extensive femoral involvement (> 70–80% of bone length), reconstruction becomes particularly challenging. The two main limb-preserving options are total femoral replacement (TFR) and biological reconstruction using long autografts or allograft–prosthesis composites. While these methods restore limb continuity, both have important limitations. TFR is associated with high complication and revision rates, including joint instability, implant failure, and functional impairment [4–6]. Similarly, long-segment frozen autografts (≥ 15 cm) are prone to fracture, delayed union, and structural failure [7, 8]. Although allograft–prosthesis composites may combine biological and mechanical advantages, their use is limited by graft availability and complication risk [9–11]. Thus, both current extremes—full-length megaprosthesis and biological reconstruction—impose significant surgical morbidity and mechanical risk.

To address these limitations, we propose a hybrid reconstruction technique combining a liquid nitrogen-treated autograft with a modular megaprosthesis (LN-MP). This technique is indicated in cases of extensive bone tumors where the remaining native bone, after tumor resection, is insufficient for stable prosthetic stem fixation. The resected tumor-bearing segment is treated with liquid nitrogen and reimplanted to restore bone length, allowing reconstruction of only one joint with a megaprosthesis and avoiding the need for TFR.

Unlike the conventional autograft–prosthesis composite techniques that rely on long devitalized grafts [12, 13], our approach uses a shorter cryo-treated autograft, which reduces graft-related complications. Our method offers the dual advantages of early postoperative mobilization and functional recovery, as well as preservation of one joint and mechanical benefits of the retained autograft segment.

The purpose of this study was to evaluate whether limb-salvage reconstruction using LN-MP could provide oncological outcomes comparable to TFR in patients with extensive femoral tumors and insufficient residual bone for conventional segmental megaprosthetic fixation. Secondary objectives included assessment of functional outcomes, perioperative characteristics, and bone-healing following LN-MP reconstruction.

## Material and method

### Study subjects

This study was conducted using retrospective data and was exempt from ethical review in accordance with the institutional policies. The study analyzed the clinical data of 28 patients with malignant bone tumors of the femur who were admitted to our hospital between March 2020 and November 2024.

### Inclusion and exclusion criteria

The inclusion criteria were as follows: (1) primary malignant bone tumors of the femur, (2) a residual host bone segment adjacent to the joint measuring ≤ 5 cm after tumor resection, and (3) reconstruction using either a TFR or a LN-MP.

The exclusion criteria were as follows: (1) bone metastases, (2) non-first-time surgical patients, (3) patients who underwent reconstruction for non-tumor factors. According to the above inclusion and exclusion criteria, 28 patients were included in this study cohort.

### Treatment selection and surgical planning

The indications for LN-MP and TFR were similar. All patients had extensive femoral tumors with limited residual host bone, making conventional proximal or distal femoral megaprosthetic reconstruction difficult or impossible.

During the early study period, TFR represented the standard limb-salvage procedure at our institution for these patients. After the surgical team gained experience with liquid nitrogen-treated autograft reconstruction, the LN-MP technique was progressively adopted as an alternative joint-preserving strategy.

**Fig. 1:**
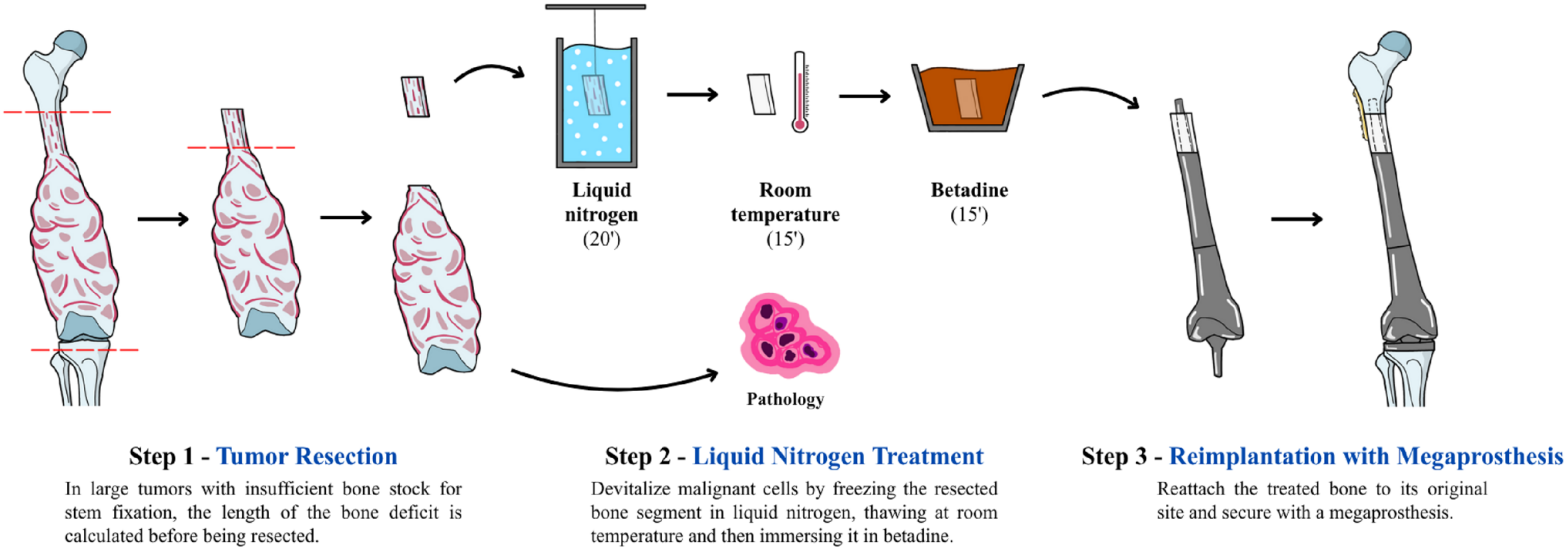
Illustration of the reconstruction procedure using a liquid nitrogen-treated autograft combined with a megaprosthesis

Preoperative planning was performed using plain radiographs, computed tomography, and magnetic resonance imaging. A residual host bone segment shorter than approximately 11 cm was considered a strong indication for LN-MP reconstruction because 11 cm corresponded to the standard stem length available in our modular megaprosthetic system. The expected residual host bone length after tumor resection was measured, and the required length of the liquid nitrogen-treated autograft was determined individually. Factors considered included residual host bone length, stem length requirements, cortical bone preservation within the tumor-bearing segment, and overall mechanical stability of the reconstruction (Fig. 1).

### Statistical analysis

All data were analyzed using JASP version 0.19.3. Continuous variables were expressed as mean ± standard deviation (SD) and categorical variables as frequencies and percentages. Between-group comparisons of continuous data (e.g., operative time, blood loss, MSTS score) were performed using the independent-samples t-test after confirming normal distribution with the Shapiro–Wilk test. When assumptions of normality were not met, the Mann–Whitney U test was applied. Categorical variables (e.g., stage, local recurrence rates) were compared using the chi-square test or Fisher’s exact test as appropriate. Correlations between graft length and time to union were evaluated using Pearson’s correlation coefficient (r) with corresponding 95% confidence intervals (CI). A two-tailed p value < 0.05 was considered statistically significant for all tests.

## Results

### Patient demographics

The study included 28 patients (age range 8–24 years) with primary malignant femoral tumors. The LN-MP group (n = 10) was significantly younger than the TFR group (n = 18) (11.6 ± 4.8 vs. 16.4 ± 3.8 years, p = 0.006). Sex distribution and disease characteristics were comparable between groups (Table 1).

In the LN-MP group, histology included osteosarcoma in 9 patients (90%) and Ewing sarcoma in 1 (10%). Tumor location was distal femur in 9 cases (90%) and proximal femur in 1 case (10%).

In the TFR group, osteosarcoma was present in 16 patients (88.9%) and Ewing sarcoma in 2 (11.1%). Tumor location was distal femur in 16 cases (88.9%) and proximal femur in 2 cases (11.1%).

**Table 1:** Demographic characteristics of study participants

Characteristic	LN-MP (n = 10)	TFR (n = 18)	p-value
Age, years (mean ± SD)	11.6 ± 4.8	16.4 ± 3.8	0.006
Sex, n (%)			0.434
Male	3 (30.0)	9 (50.0)	
Female	7 (70.0)	9 (50.0)	
Tumor location			
Proximal	1 (10.0)	2 (11.1)	
Distal	9 (90.0)	16 (88.9)	
Disease classification, n (%)			0.388
Conventional osteosarcoma	9 (90.0)	16 (88.8)	
Ewing	1 (10.0)	2 (11.8)	
Enneking stage, n (%)			0.434
IIB	7 (70.0)	9 (50.0)	
III	3 (30.0)	9 (50.0)	

### Perioperative characteristics of patients

The LN-MP group had shorter operative time (253.0 ± 59.6 vs. 310.6 ± 47.5 min, p = 0.009) and lower intraoperative blood loss (500.0 ± 226.1 vs. 927.8 ± 433.6 mL, p = 0.008) compared with TFR.

Length of hospital stay was slightly shorter in the LN-MP group (8.5 [6.0–9.0] vs. 9.0 [6.0–16.0] days, p = 0.032), while follow-up duration was similar between groups (p = 0.339) (Table 2).

**Table 2:** Perioperative characteristics of patients

Characteristic	LN-MP (n = 10)	TFR (n = 18)	p-value
Duration of operation, min	253.0 ± 59.6	310.6 ± 47.5	0.009
Intraoperative blood loss, mL	500.0 ± 226.1	927.778 ± 433.6	0.008
Length of stay, days (median, IQR)	8.5 (6.0–9.0)	9.0 (6.0–16.0)	0.032
Follow-up time, months (median, IQR)	16.5 (6.0–29.0)	13.5 (5.0–65.0)	0.339

### Surgical outcomes

Limb-salvage reconstruction using LN-MP demonstrated a higher continuous disease-free (CDF) survival rate compared with TFR (90.0% vs. 44.4%, p = 0.058). Although this difference did not reach statistical significance, it indicated a trend toward improved oncologic control in the LN-MP group. Local recurrence occurred in 1 patient (10.0%) in the LN-MP group and 5 patients (27.8%) in the TFR group.

Functional outcomes were significantly better in the LN-MP group, with higher MSTS scores compared with TFR (27.0 ± 2.1 vs. 24.4 ± 2.2, p = 0.014) (Table 3).

**Table 3:** Surgical Outcomes

Outcome/complication	LN-MP (n = 10)	TFR (n = 18)	p-value
Oncological outcome, n (%)			0.058
CDF	9 (90.0%)	8 (44.4%)	
AWD	0 (0%)	2 (11.1%)	
DOD	1 (10.0%)	8 (44.4%)	
Intraoperative complications	None	None	
Local recurrence, n (%)	1 (10.0%)	5 (27.8%)	0.375
MSTS score, mean ± SD	27.0 ± 2.1	24.4 ± 2.2	0.014

*CDF* clinically disease-free, *AWD* alive with disease, *DOD* death of disease

### Bone-related outcomes in the LN-MP group

**Fig. 2:**
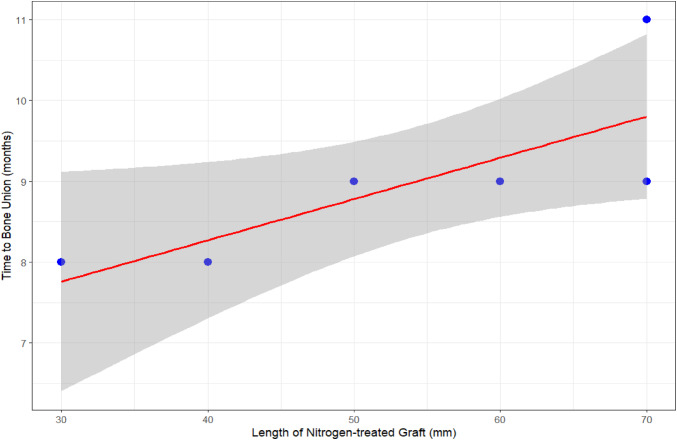
Correlation between graft length and time to union

Among the 10 patients in the LN-MP group, 7 (70.0%) achieved successful bone union at the graft–host junction. The mean time to union was 9.0 ± 1.0 months (range 8–11 months). A significant positive correlation was identified between graft length and time to union (r = 0.77, 95% CI 0.04–0.96, p = 0.042) (Table 4; Fig. 2).

**Table 4:** Bone-related outcomes in the LN-MP group

Parameter	LN-MP (n = 10)
Bone union achieved, n (%)	7/10 (70.0%)
Time to bone union, months (mean ± SD)	9.0 ± 1.0 (range 8–11)
Length of nitrogen-treated graft, mm	49.0 ± 15.2 (30–70)
Length of resected bone, mm	216.9 ± 41.3 (131–270)
Residual host bone length, mm	85.0 (55–100)

## Discussion

The findings suggest that LN-MP provides oncologic outcomes comparable to TFR. In addition, LN-MP was associated with better functional outcomes and acceptable rates of bone union.

### Disease-free and overall survival

Accumulating evidence indicates that modern “recycle and reimplant” techniques—including liquid nitrogen treatment, pasteurization, and irradiation—do not increase local recurrence compared with standard megaprosthetic reconstruction [14–17]. A recent systematic review reported a local recurrence rate of 8.7% within 4 years for liquid nitrogen-treated autografts, which compares favorably with rates of 15–30% reported for megaprosthesis-based reconstruction [18–21]. Experimental studies have also proposed that cryo-treated grafts may induce anti-tumor immune responses through antigen exposure during the freeze–thaw process, although the clinical relevance of this mechanism remains to be fully established [22, 23].

Because of the small sample size, a single recurrence event represented 10% of the LN-MP cohort. In addition, our study specifically included patients with extremely large femoral tumors involving most of the femoral length, a subgroup that may have a higher risk of local failure than the broader population of extremity bone sarcomas. Therefore, the recurrence rates reported here likely reflect the advanced nature of the disease rather than the reconstructive technique itself.

### Functional outcomes

**Fig. 3:**
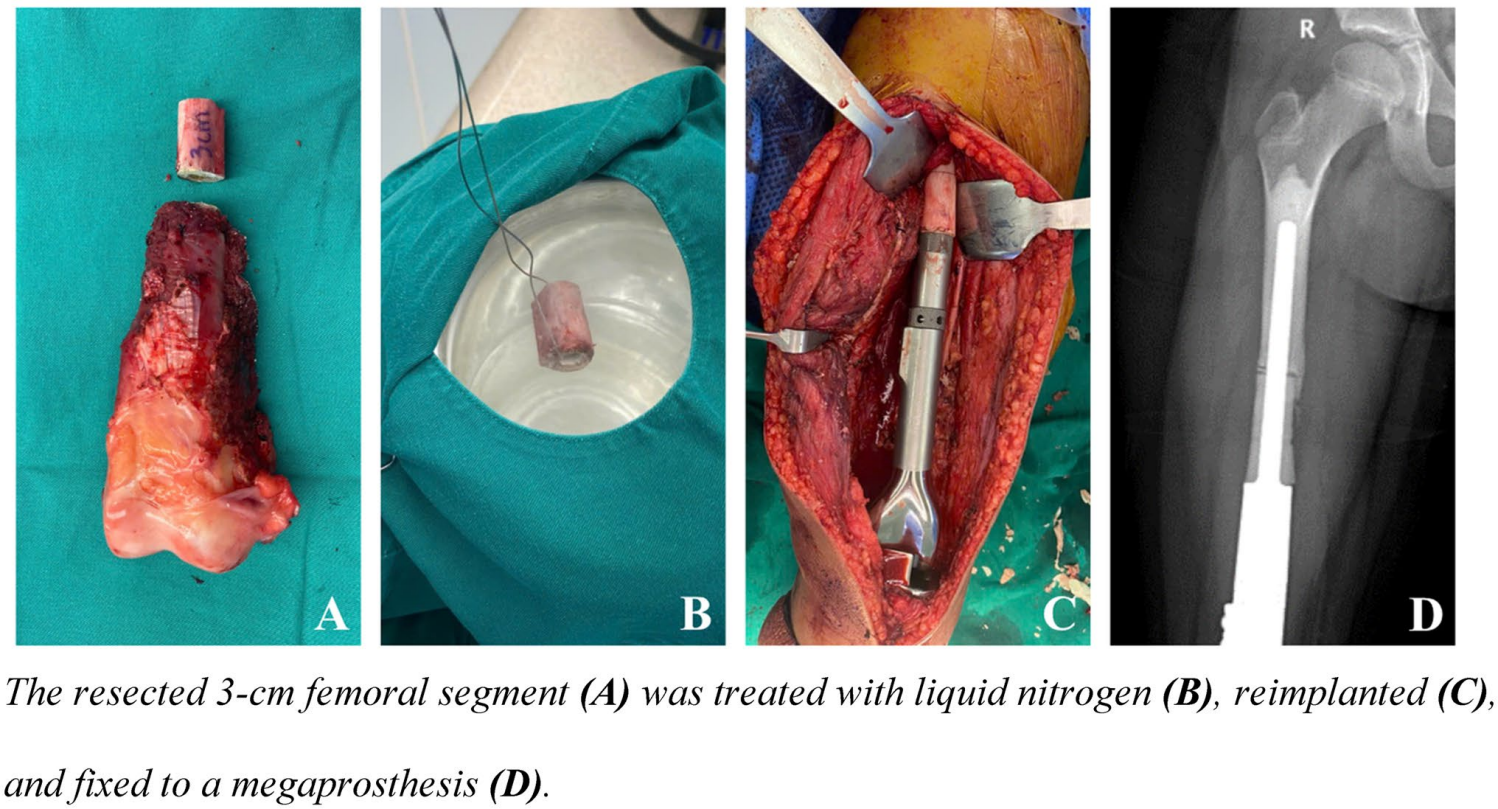
Intraoperative and postoperative image demonstrating reconstruction of a distal femoral tumor using a liquid nitrogen-treated autograft combined with a megaprosthesis

**Fig. 4:**
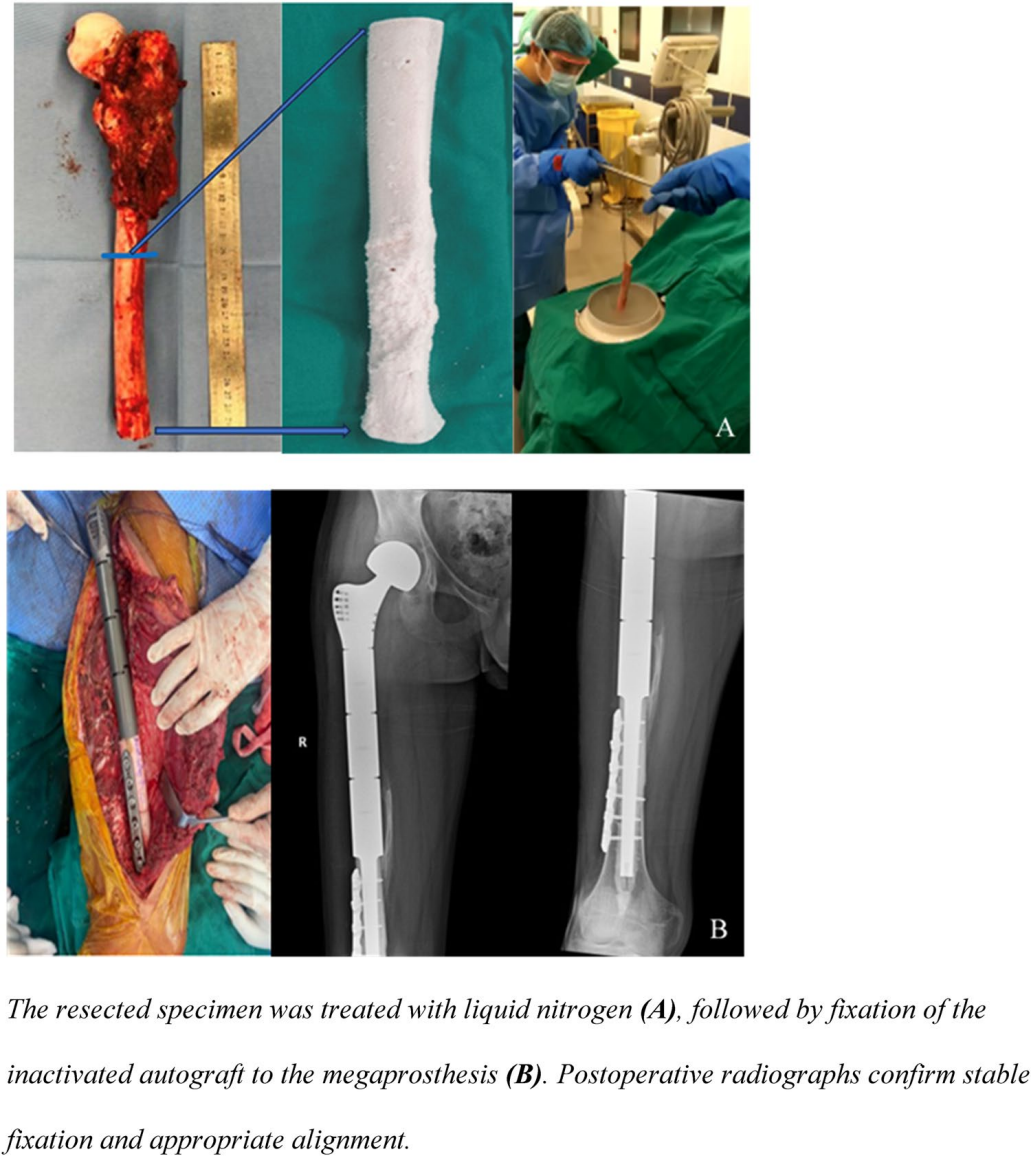
Intraoperative and postoperative radiographs demonstrating reconstruction of a proximal femoral tumor using a liquid nitrogen-treated autograft combined with a megaprosthesis

Patients treated with LN-MP achieved significantly higher MSTS scores than those undergoing TFR. Retaining the original hip or knee joint and surrounding soft-tissue attachments may allow for more physiological biomechanics, which could contribute to improved functional recovery (Figs. 3 and 4).

Recent advances in personalized oncologic reconstruction have expanded the possibilities for joint preservation in patients with limited residual bone stock. Custom-made endoprostheses, and patient-specific 3D-printed implants have demonstrated encouraging outcomes in situations where conventional stem fixation is difficult. However, in cases with extremely extensive femoral involvement, the challenge extends beyond joint preservation alone and includes obtaining sufficient fixation length to ensure long-term mechanical stability. The rationale of the LN-MP technique is to restore additional biological bone stock through reimplantation of a liquid nitrogen-treated autograft, thereby permitting the use of a longer prosthetic stem while preserving one native joint.

### Intraoperative and early postoperative outcomes

Operative time was significantly shorter in the LN-MP group compared with TFR, likely reflecting reduced surgical complexity, fewer osteotomies, and reconstruction of only one joint. The mean operative time for TFR in our cohort (~ 5.2 h) is consistent with previously reported ranges of 4–6 h [6, 24, 25].

Both cohorts required a similar duration of postoperative care, typically 1–2 weeks. This may be related to cautious rehabilitation protocols following LN-MP, where early weight-bearing is often restricted to reduce graft-related complications. As a result, the reduction in operative time and blood loss did not translate into earlier discharge.

From an economic perspective, LN-MP reconstruction was associated with lower implant costs. Although overall hospital stay and bed-related costs were comparable, the reduced implant expense may represent a practical advantage, particularly in resource-limited settings.

### Bone union outcomes in liquid nitrogen-treated autograft

**Fig. 5:**
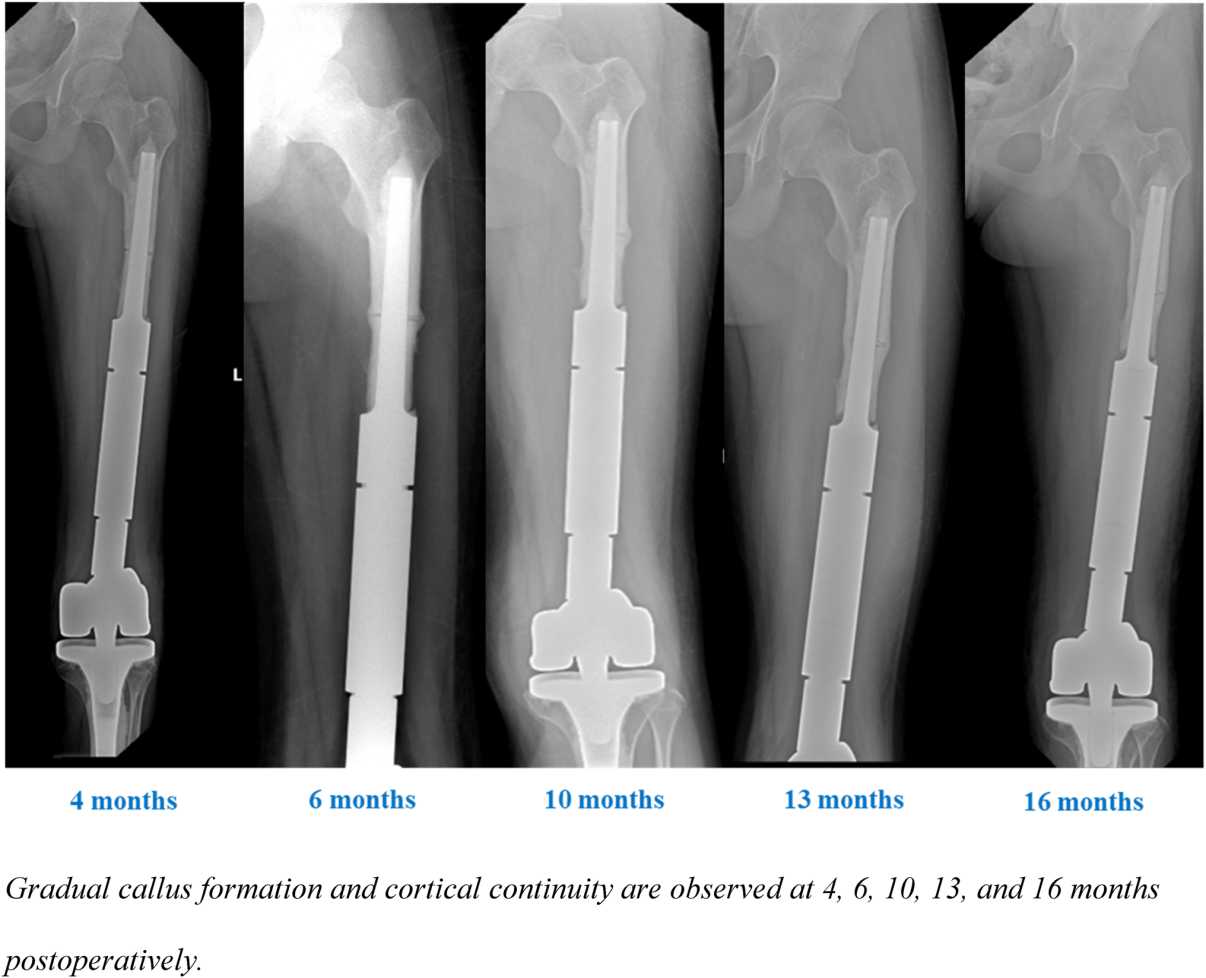
Serial radiographs demonstrating progressive bone union after reconstruction using a liquid nitrogen-treated autograft combined with a megaprosthesis

In our series, LN-MP reconstruction achieved graft–host union at a mean of approximately 9 months, consistent with previous reports [26, 27]. However, the observed union rate of 70% is at the lower end of published ranges. This may be related to differences in surgical technique, as prior studies frequently used pedicle freezing methods that preserve partial vascularity, whereas our study employed free grafts with no intrinsic blood supply at either end (Fig. 5).

A significant positive correlation was observed between graft length and time to union, indicating that longer grafts required more time to heal. This finding is consistent with previous studies [28, 29]. Biologically, longer grafts have reduced revascularization capacity, while mechanically they are subjected to greater stress at the graft–host junction, which may impair healing.

These findings suggest that graft length is an important factor influencing union, and that shorter graft segments may achieve more reliable and timely integration.

This study has several inherent limitations. First, its retrospective design may introduce selection bias, and the allocation into two groups was not randomized. The sample size was small, reflecting the rarity of large-volume osteosarcoma. Second, although structural allograft reconstruction represents another viable biological reconstruction method, this option was not available at our institution because of the absence of a bone bank and the difficulty of obtaining size-matched allografts, particularly in pediatric patients. Third, oncological outcomes are affected by multiple factors beyond the reconstruction method itself, including tumor stage, biological behavior, response to chemotherapy, surgical margins, and metastatic status. Therefore, the present study was not designed to determine whether reconstruction type independently influences oncological control, and any observed survival differences should be interpreted cautiously. Finally, complications such as aseptic loosening of the endoprosthesis or graft-related fracture and osteolysis are known to increase over longer follow-up periods. As our current follow-up remains relatively short, ongoing observation is necessary to assess 10-year outcomes for both patient survival and graft longevity.

## Conclusions

In conclusion, LN-MP may serve as a joint-preserving alternative to total femoral replacement. n the present cohort, LN-MP demonstrated favorable functional outcomes and satisfactory bone union, while oncological outcomes appeared comparable to those observed after TFR. Given the retrospective design, small sample size, and limited follow-up, these findings should be interpreted cautiously and require validation in larger studies.

## Data Availability

The datasets analysed during the current study are available from the corresponding author on reasonable request.

## Abbreviations

APC: Allograft–prosthesis composite
LN-MP: Liquid nitrogen autograft–megaprosthesis
TFR: Total femur replacement
MSTS: Musculoskeletal Tumor Society score [34]
